# Medical Education

**Published:** 1901-09-07

**Authors:** 


### Medical Education.

When a young man has decided to enter the medical profession, and when he has gone so far as to consult the Dean of some medical school as to the course he shall pursue he will have at his disposal expert advice in regard to every step that he proposes to take. To such, then, no word from us is required. But to many who have not gone so far as this, and are still hovering on the brink the whole scheme of medical education is to a large extent a *terra incognita*, and to these some words of advice and some information regarding degrees, diplomas, and course of study will, we think, be useful. There are many reasons why anyone who is desirous of entering the medical profession should look carefully ahead and exercise some discrimination in deciding upon the qualification which he will take, and the school at which he will be educated.

### University Degrees.

For many years past it has been becoming increasingly clear that even in ordinary general practice a man without a University degree is at a disadvantage, while if he wishes to practise as a consultant on the medical side a degree is absolutely essential. It is true that in professional circles the ordinary M.B. or M.B., C.M., which is granted by so many of the Universities is not regarded as in any way a mark of overwhelming superiority. But with the public it is different. People do not care to enter into minutiæ in regard to the course of study required for a diploma and a degree. It is no use arguing with them that to be a member of a Royal College of England is quite as honourable and gives quite as good a professional position as the possession of a provincial degree. All they know, and all they care to know, is that the University graduate can put certain letters after his name which others cannot do, and nothing will persuade them that the man who can append the M.D. to his signature is not *prima facie* the better man. Hence the great importance of so arranging one's course of study as to come out at the end not merely Mr. Jones, Surgeon, but Dr. Jones! No man knows at the beginning of his career in what direction his tastes will take him. Still less can he guess what sort of openings may occur, or towards what line of practice he may drift under the influence of apparently fortuitous circumstances, and it has been an extreme disappointment to many to find themselves debarred by want of a suitable qualification from openings which have seemed, however doubtfully, to lead to fortune. Thus in the great majority of cases a University degree should be aimed at, and this involves a good deal. In most Universities it involves residence, and in some it involves a rather stiff matriculation examination. Take for example the London student. He wants an M.D. Where is he to get it? We cannot speak positively about the future action of the University of London, but in regard to the present condition of affairs, which will hold good for at least this year, it may be said not only that the professional examinations are difficult, although perhaps not so difficult as some imagine, but that the matriculation stands in the way of all who have not specially prepared themselves for it. The same is true, but in a different degree, in regard to the arts examinations which other Universities require from their students. They are none of them inordinately difficult, but they all require that the work of the last year or so of school life should have been specially arranged in accordance with the demands of the particular examination selected. It is then of the utmost importance that parents should look ahead in regard to all these matters, and instead of waiting until their boys leave school before deciding what shall be done with them, should make up their minds earlier so that before the discipline of school life has been relaxed they shall have obtained a good preparation for the particular examination required at the medical school selected, which, as we have already said, should in most cases be, if possible, a University.

### College Diplomas.

For general practice no doubt a degree from a University is enough. But other things have to be thought of. If a student turns out well he may aspire to something higher than general practice, and if he wishes to become physician or surgeon to a large hospital he will probably find himself barred from his goal unless he can become a Member of the College of Physicians or a Fellow of the College of Surgeons as the case may be. Hence it is worth every man's while, even though he take a degree, to become also a Member of the Royal College of Surgeons and a Licentiate of the Royal College of Physicians of London. This is done by passing a series of examinations which go by the name of the "Conjoint," being arranged by a board appointed by the two Colleges. This double diploma is itself a full qualification admitting to the Register, but its great advantage is that it forms the first stage, the necessary preliminary to obtaining those higher diplomas which are required for most hospital appointments. But the Conjoint can be prepared for at any medical school either in England, Scotland, or Ireland, so that in the choice of a school one comes back to the old point, that if possible the one selected should either be a University or should be so attached to a University as to enable the student without difficulty to obtain a degree at the end of his time.

### Place of Study.

The advantages of studying medicine in London are undoubted, and if the student has been sufficiently well educated and has a sufficiently good head to be able with comparative ease to matriculate at the University of London he is pretty sure to be able to pass the further examinations, and for such a student we can have no hesitation in advising that he should enter at one of the London schools and work steadily for the London M.B. and the Conjoint. If, however, he should find a difficulty in passing the matriculation he may enter either at Cambridge or Durham, especially the former. The advantages of Cambridge are manifold. The scientific portions of the curriculum are admirably taught there; then it has for so long a time been the custom for the Cambridge students to do their hospital work in London (and the same is the case with the students from Oxford) that there is nothing provincial about its degrees; and in addition to all this there is no doubt that the degrees of the two old well-recognised English universities occupy a position in men's minds which is special to themselves. The other Universities—Durham, the Victoria, and Birmingham—may be regarded as local institutions provided for the accommodation of the several districts served by them, and as the colleges connected with each of them offer a complete medical education it is customary for their students to remain with them to the end. A good many men, however, go to Durham for only a part of their time, the attraction to this University being that only one year of residence is required, so that when that is over it is easy to come to London for the rest of the time.

We must not, however, pass over the Scottish Universities, which are admirable teaching institutions. The whole system is somewhat different from what obtains in most English schools, but they turn out very good men and report has it that the course can be gone through and the degree obtained in Scotland upon much cheaper terms than is the case in England. It is probable, however, that this is largely due to readiness of the Scottish student to live in a less expensive manner than is commonly adopted by students in England. Those, then, whose means are small should carefully consider the Universities of Scotland. It is always important that a student should feel on terms of equality with his fellows, and it is far easier and more pleasant to live frugally where others are doing the same than to be the odd man among a crowd well supplied with the good things of this world. Study at any of the Scottish schools qualifies for the "Conjoint."

Our advice then to the student who is not tied by local circumstances is—London, if possible; if not, Cambridge or Durham for the first part of the curriculum, doing the hospital work in London; and if not this, then Scotland, always endeavouring, whatever the school selected, to come out at the end of the course the possessor of a degree and the "Conjoint." If this, which is the best plan, cannot be followed, then either the Conjoint or a degree may be taken separately; or, failing that, the student may become a Licentiate of the Society of Apothecaries, which at least will place him on the Register and enable him to practise. Once on the Register all are alike in the eyes of the law, whatever their qualifications. But they are not all alike in the eyes of the public nor in those of the profession, and thus it is an immense advantage to start life with a good degree.

